# P-1406. Racial Disparities in HIV Prevalence and Complications in Hospitalized Patients

**DOI:** 10.1093/ofid/ofae631.1581

**Published:** 2025-01-29

**Authors:** Armin Safdarpour, Chawki Harfouch, Chukwuemeka Umeh

**Affiliations:** KPC Hemet Global Medical Center, Menifee, California; KPC Hemet Global Medical Center, Menifee, California; KPC Hemet Global Medical Center, Menifee, California

## Abstract

**Background:**

Racial and ethnic disparities in HIV care and outcomes are well known. Multiple causes have been reported including inequalities in access to care and pre-exposure prophylaxis (PrEP), housing instability, lack of antiretroviral insurance coverage, and economic disadvantages. We aim to evaluate the disparities in HIV prevalence and outcomes in hospitalized patients.

**Methods:**

We analyzed the 2020 United States National Inpatient Sample (NIS) data of all patients with a diagnosis of HIV admitted inpatient. The NIS database is the largest publicly available database of patients hospitalized in the United States and contains approximately 20% stratified samples of all hospital discharges in the United States.

**Results:**

There were 38,666 admissions with a diagnosis of HIV in 2020, of which 30.3% were females. 51.6% of the HIV patients were Blacks, 30% Whites, 13.7% Hispanics, 0.7% Asians, 0.6% Native Americans, and 3.4% others. The mean age was 50.8 years, and the mean length of stay was 6.4 days. In the bivariate analysis, the Hispanics were more likely compared to others to have Kaposi Sarcoma (1.8% vs. 1.1%, p< 0.001), cryptococcal meningitis (1.5% vs. 0.6%, p< 0.001), disseminated coccidioidomycosis (0.2% vs 0.0%, p< 0.001), hepatitis C (6.7% vs. 5.3%, p< 0.001), Covid-19 infection (7.9% vs. 4.9%, p< 0.001) and pneumocystis jiroveci pneumonia (3.2% vs. 2.3%, p< 0.001). The multivariate analysis showed no statistically significant racial or ethnic difference in all-cause mortality (p=0.17). However, patients in the highest income quartile had significantly lower all-cause mortality compared to those in the lowest quartile after adjusting for variables including race, ethnicity, and HIV complications (OR 0.69, 95% CI 0.54-0.89, p=0.004).

**Conclusion:**

Our study showed that the prevalence of HIV is still disproportionally high in Blacks. While about 16% of all patients in our dataset are Black, about 52% of the HIV patients are Black. However, Hispanic patients appear to have more HIV complications. Cultural appropriate interventions to prevent HIV acquisition, increase HIV screening, and early initiation of treatment in racial and ethnic minorities are warranted.

Mining a national database can provide insights into morbidity and mortality trends in patients living with HIV.

**Disclosures:**

**All Authors**: No reported disclosures

